# Host–Microbiome Crosstalk in Chronic Wound Healing

**DOI:** 10.3390/ijms25094629

**Published:** 2024-04-24

**Authors:** Mara Mădălina Mihai, Beatrice Bălăceanu-Gurău, Ana Ion, Alina Maria Holban, Cristian-Dorin Gurău, Marius Nicolae Popescu, Cristina Beiu, Liliana Gabriela Popa, Mircea Ioan Popa, Cerasella Cristiana Dragomirescu, Mădălina Preda, Alexandru-Andrei Muntean, Ioana Sabina Macovei, Veronica Lazăr

**Affiliations:** 1 Department of Oncologic Dermatology, “Elias” Emergency University Hospital, “Carol Davila” University of Medicine and Pharmacy, 020021 Bucharest, Romania; mara.mihai@umfcd.ro (M.M.M.); cristina.beiu@umfcd.ro (C.B.); liliana.popa@umfcd.ro (L.G.P.); 2 Clinic of Dermatology, “Elias” Emergency University Hospital, 011461 Bucharest, Romania; 3 Research Institute of the University of Bucharest, Department of Botany-Microbiology, Faculty of Biology, University of Bucharest, 050663 Bucharest, Romania; alina_m_h@yahoo.com (A.M.H.); veronica.lazar2009@gmail.com (V.L.); 4 Orthopedics and Traumatology Clinic, Clinical Emergency Hospital, 014451 Bucharest, Romania; gurau_dorin@yahoo.com; 5 Department of Physical and Rehabilitation Medicine, “Elias” Emergency University Hospital, “Carol Davila” University of Medicine and Pharmacy, 020021 Bucharest, Romania; marius.popescu@umfcd.ro; 6 Clinic of Physical and Rehabilitation Medicine, “Elias” Emergency University Hospital, 011461 Bucharest, Romania; 7 Department of Microbiology, “Cantacuzino” Institute, “Carol Davila” University of Medicine and Pharmacy, 020021 Bucharest, Romania; mircea.ioan.popa@umfcd.ro (M.I.P.); ceraseladragomirescu@yahoo.com (C.C.D.); muntean.alex@gmail.com (A.-A.M.); 8 Cantacuzino National Military Medical Institute for Research and Development, 050096 Bucharest, Romania; madalina.prd@gmail.com (M.P.); ioana.s.macovei@gmail.com (I.S.M.); 9 Department of Microbiology, Parasitology and Virology, Faculty of Midwives and Nursing, “Carol Davila” University of Medicine and Pharmacy, 020021 Bucharest, Romania

**Keywords:** host immunity, host–pathogen communication, host–pathogen interplay, biomarker, bacterial signaling, microbial biofilms, chronic wounds, chronic ulcers, prebiotics, probiotics

## Abstract

The pathogenesis of chronic wounds (CW) involves a multifaceted interplay of biochemical, immunological, hematological, and microbiological interactions. Biofilm development is a significant virulence trait which enhances microbial survival and pathogenicity and has various implications on the development and management of CW. Biofilms induce a prolonged suboptimal inflammation in the wound microenvironment, associated with delayed healing. The composition of wound fluid (WF) adds more complexity to the subject, with proven pro-inflammatory properties and an intricate crosstalk among cytokines, chemokines, microRNAs, proteases, growth factors, and ECM components. One approach to achieve information on the mechanisms of disease progression and therapeutic response is the use of multiple high-throughput ‘OMIC’ modalities (genomic, proteomic, lipidomic, metabolomic assays), facilitating the discovery of potential biomarkers for wound healing, which may represent a breakthrough in this field and a major help in addressing delayed wound healing. In this review article, we aim to summarize the current progress achieved in host–microbiome crosstalk in the spectrum of CW healing and highlight future innovative strategies to boost the host immune response against infections, focusing on the interaction between pathogens and their hosts (for instance, by harnessing microorganisms like probiotics), which may serve as the prospective advancement of vaccines and treatments against infections.

## 1. Introduction

Chronic wounds (CWs), especially venous and arterial leg ulcers (VLU, ALU), neuropathic leg ulcers, diabetic foot ulcers (DFU), pressure sores, and non-healing surgical wounds represent a significant burden to the healthcare systems worldwide [1,2,3].

Wound healing is a complex, highly integrated process, mediated by leukocytes, platelets, lymphocytes, fibroblasts, macrophages, vascular smooth muscle cells, endothelial cells, and keratinocytes [4,5,6,7]. The normal healing process involves clot formation, inflammation, re-epithelialization, angiogenesis, granulation tissue formation, wound contraction, scar development, and tissue remodeling, while the immune system seems to be responsible for the regulation of all the aforementioned processes [1,4,5,6,7,8,9,10]. Nevertheless, there is also a release of diverse biomolecules including the following: transforming growth factor-β (TGF-β), cytokines, chemokines, matrix metalloproteinases (MMPs), tissue inhibitors of MMPs (TIMPs), elastase, urokinase plasminogen activator, fibrin, collagen, and albumin [3,7,11].

The pathogenesis of CWs involves a multifaceted interplay of biochemical, immunological, hematological, and microbiological interactions. CWs share an essential common feature—persistent, low-grade inflammation that impedes progress towards further proliferation [7,8,12,13]. Delayed healing is a direct consequence of the alteration of physiological wound closure events, together with microbial colonization, infection, and high levels of exudate [1,4,7]. Different from normal healing wounds, chronic ulcers seem to have impaired cellular recruitment, cell activation, angiogenesis, as well as extracellular matrix (ECM) remodeling [12]. The release of chemokines (IL-1, IL-2, tumor necrosis factor—TNF) by macrophages and the migration of T lymphocytes to the wound bed play major roles in healing [1,4].

The polymicrobial contamination of long-standing, non-healing wounds has been well established through solid evidence throughout the years [8,14,15,16]. The long-term presence of a wound provides a favorable environment for both skin commensal bacteria (part of the cutaneous microbiome) and pathogens to colonize, grow, and invade the underlying tissues [15,17]. It was found that the interplay between commensal bacteria and host skin cells during the normal healing stages may modulate the innate immune reaction [17]. Nevertheless, it was proven that microorganisms also play a significant part in delayed wound healing, especially through the development of biofilms [2]. A total of 60% of CWs are colonized by biofilm-forming bacteria, with two of the most common species being *Staphylococcus aureus* and *Pseudomonas aeruginosa* [18].

**Figure 1:**
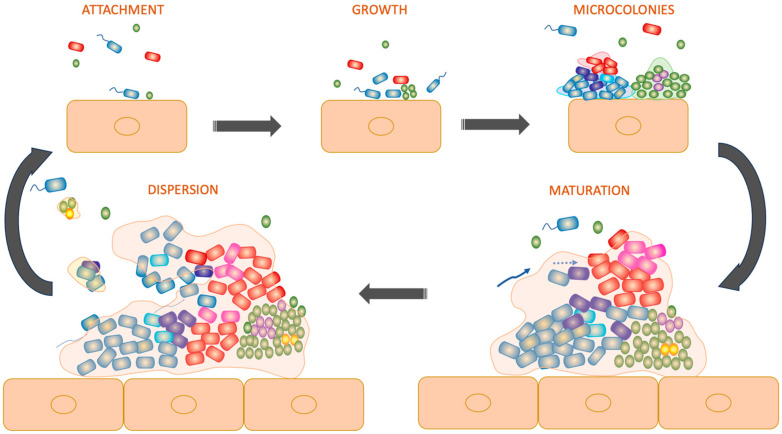
Graphic representation of biofilm formation within a wound, starting from the attachment of bacteria and followed by growth, the formation of microcolonies, the maturation phase, and finally, dispersion.

Considered a significant virulence trait, biofilm development enhances microbial survival and pathogenicity and has various implications on the development and management of CWs [18]. Biofilms induce a prolonged suboptimal inflammation in the wound microenvironment, associated with delayed healing [18]. Risk factors for biofilm development include chronic venous insufficiency, diabetes mellitus, malnutrition, edema, cancer, and local repetitive trauma [18]. When dealing with biofilm infections, it is of utmost importance to consider wound bed preparation as a crucial part in the therapeutic approach of CWs, as well as sharp wound debridement for an effective removal or reduction in biofilm formation (Figure 1) [18].

One approach to achieve information on the mechanisms of disease progression and therapeutic response is the use of multiple high-throughput ‘OMIC’ modalities (genomic, proteomic, lipidomic, metabolomic assays), facilitating the discovery of potential biomarkers for wound healing, which may represent a breakthrough in this field and a major help in addressing delayed wound healing. The host–microbiome crosstalk in the context of CW infections is a crucial determinant in the complex process of wound healing, and its study may be useful to overcome longstanding controversies on pathogenicity and to discover innovative treatment strategies.

In this review article, we aim to summarize the current progress achieved in host microbiome molecular interactions in the spectrum of CWs healing and to highlight future innovative strategies to boost the host immune response against infections, focusing on the interaction between pathogens and their hosts (for instance, by harnessing beneficial microorganisms like probiotics), which may serve as a prospective advancement of the vaccines and treatments against infections.

## 2. The Composition of Chronic Wound Microbiome

Infection is regarded as one of the crucial factors in generating and maintaining long-standing wounds [18]. In most studies regarding the microbiology of wound healing, bacteria were cultured from infected CWs [19]. However, microbial colonization influences wound healing even in the absence of an active infection [19]. Nevertheless, it was suggested that the skin microbiome may impact the wound healing process in multiple ways, not only by variations in bacterial load [19]. Bacterial colonization is believed to be involved in driving chronic inflammation, wound chronicity, and the development of fibrosis [20]. In CWs, there is an increased state of activation of host cells influenced by the microbiota which may lead to negative effects on wound regeneration [20].

Moreover, the development of polymicrobial biofilms disrupts the well-coordinated events of the normal healing process, playing an essential part in the pathogenesis of impaired wound healing [21].

Wound microbiome may be studied using traditional culture-dependent methods (with multiple limitations) and more advanced culture-independent methods such as next-generation sequencing techniques. The amplification and sequencing of various regions of the microbial 16S rRNA for phylogenetic analysis aids in investigating the bacterial composition of a wide range of CWs, including venous insufficiency ulcers, DFUs, and pressure ulcers [19]. Published reports of sequence analyses of the bacteria from CWs showed that, as compared to the normal skin microbiota, CWs present more Gram-negative bacilli, anaerobes, and Gram-positive cocci [22]. Moreover, CWs showed less commensals such as *Cutibacterium* [22]. The major bacterial species are represented by *Staphylococcus*, *Streptococcus*, *Pseudomonas*, *Corynebacterium,* and anaerobes [19,23,24].

In a clinical observational study from 2016 by Wolcott et al., on 2963 patients with chronic wound specimens, the results obtained by 16S rDNA pyrosequencing showed that *Staphylococcus* was the most commonly encountered genera, with *Staphylococcus aureus* and *Staphylococcus epidermidis* identified as the most abundant Gram-positive bacteria in chronic ulcers [25]. *Staphylococcus epidermidis* seemed to be more prevalent in DFUs, while *Pseudomonas aeruginosa* showed a higher relative abundance in all types of CWs demonstrating biofilm formation [25].

An experimental study from 2019 emphasized the co-occurrence of anaerobes in human CWs by 16S ribosomal RNA gene sequencing [13]. Investigations showed the following results: the group of obligate anaerobes identified by taxonomic analysis was represented by the Clostridia class, as well as strict anaerobes such as *Prevotella* and *Porphyromonas* [13]. Another finding was that anaerobes co-existed with commonly identified bacterial species, such as *Staphylococcus epidermidis, Staphylococcus aureus*, *Corynebacterium*, *Pseudomonas* and *Streptococcus*, in longstanding wounds and even more importantly, in some cases, they were predominant within the wound microbiota [13].

In VLUs, it was found that some of the most abundant bacterial species are *Staphylococcus*, *Pseudomonas*, *Bacteroides*, *Serratia*, *Corynebacterium*, and various anaerobes [26].

*Klebsiella pneumoniae*, *Escherichia coli*, *Morganella morganii*, *Proteus mirabilis*, *Citrobacter freundii*, and β-hemolytic streptococci of group G were strains isolated in the VLUs of patients admitted to the “Elias” Emergency University Hospital, Bucharest, Romania, over a period of 5 years.

**Figure 2:**
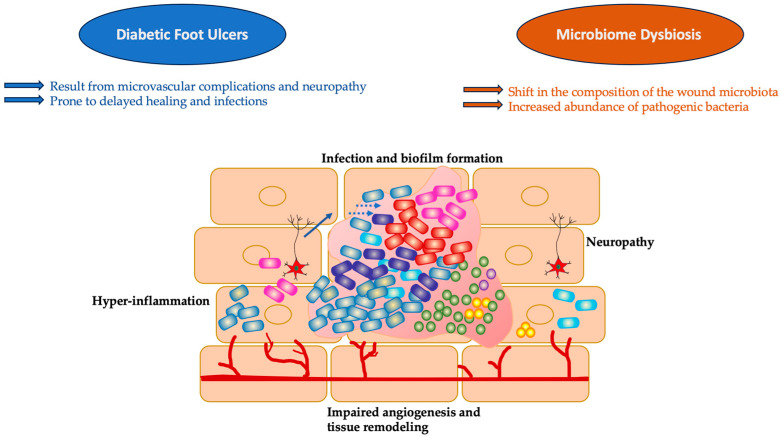
Graphic representation of the mechanisms and pathways involved in delayed wound healing in DFUs due to the microbiome crosstalk.

DFUs represent, undoubtedly, one of the most thoroughly studied fields of research with an abundance of evidence concerning the wound microbiome (Figure 2). In an experimental study from 2008 by Dowd et al. on the polymicrobial nature of DFU biofilm infections, the most prevalent genus identified was *Corynebacterium* [27]. However, *Streptococcus*, *Serratia*, *Staphylococcus*, *Bacteroides*, *Peptostreptococcus*, *Peptoniphilus*, and *Enterococcus* were also frequently observed [28].

In 2016, Smith et al. characterized the microbiome of both new and recurrent DFUs using 16S amplicon sequencing (16S AS) of samples from 20 patients who did not receive antibiotic therapy for the past three months, with the aim to identify a broad range of microbial species which may be involved in the longstanding evolution of these debilitating wounds [29]. Results showed that the most commonly detected bacterial species were *Peptoniphilus*, *Corynebacterium*, and *Anaerococcus* [29].

In 2017, Loesche et al. found, through sequencing of DNA extracted from wound debridement specimens, that in DFUs, the most abundant microorganisms were mentioned in descending order as follows: *Staphylococcus*, *Streptococcus*, *Corynebacterium*, and *Anaerococcus* [30]. This is in accordance with an experimental study from 2019 by Daeschlein et al. who demonstrated that diabetic wounds exerted disease-related changes, with the staphylococcal species dominating the wound microenvironment compared with non-diabetic wounds in which streptococcal species were abundant [31].

Concerning the composition of the pressure ulcer microbiome, Ammons et al. showed in a study from 2015 that the most predominant phylum was, firstly, Firmicutes, followed by Proteobacteria, and lastly, Actinobacteria [32]. In 2010, Smith et al. evaluated the bacterial diversity of decubitus ulcers using debridement samples from 49 pressure wounds [28]. The most common genera were found to be *Streptococcus* and *Corynebacterium*, while *Staphylococcus* was a distant third genera [28]. Overall top species were found to be represented by *Staphylococcus epidermidis*, *Corynebacterium striatum*, and *Finegoldia magna* [28]. Lastly, *Streptococcus agalactiae*, *Pseudomonas aeruginosa*, and *Corynebacterium striatum* were the most common bacterial species found within a single pressure ulcer [28].

Interestingly, the composition of the CW microbiota varies depending on the patient’s wound healing history. In new ulcers, the *Staphylococcus* spp. was exceedingly common, while *Actinomyces* was present in recurrent ulcers but was not found in recent wounds [29]. It also appeared that a higher diversity within a wound was associated with a shorter duration of the patient’s disease and better glucose control [29].

## 3. The Impact of Chronic Wound Microbiome on Healing

The wound microbiome plays a key part in tissue fibrosis and is generally considered to exert pathogenic effects on healing [20].

In a review from 2010, Percival et al. provided more in-depth insight into the role of microbial species in non-healing wounds [33] (Table 1). There were two theories proposed. In both, it is considered that bacterial biofilms occur commonly and naturally within the wounds and that they may lead to infection when the microenvironment is imbalanced due to several factors as follows: alterations in local pH, temperature, wound dressings, antimicrobial treatment, modifications in the host immune response [33]. The “specific bacterial hypothesis” states that a few species of bacteria may be disrupted from biofilms, causing infection and thus delayed wound healing [33]. The “community” or “non-specific bacterial” hypothesis states that the wound microbiota as a whole is pathogenic [33].

In vivo studies on animal models suggested that an altered composition of the wound microbiome can directly influence the healing potential [19,34]. In 2014, Canesso et al. showed that germ-free mice had an accelerated wound epithelialization, angiogenesis, and wound closure rates [35]. Moreover, decreased scarring and signals of inflammation were observed, while restoration of microbiota resulted in inflammation, cytokine production, and wound closure rates similar to that of conventional mice [35]. These results are in accordance with the hypothesis regarding the negative impact of bacteria on wounds, and that overall decreased levels of microorganisms are beneficial for wound healing [35].

In an experimental study by Wolcott et al. from 2016, answers regarding the role of the wound microbiome in the chronicity of longstanding wounds were provided [36]. In this study, wound microbiomes as units obtained from human CW were seeded onto mouse wounds produced by surgical excision, in the hope of developing chronically infected wounds resembling the chronic ones found in the original hosts [36]. The results were astonishing—81% of the mice models did develop typical chronically infected wounds [35]. Moreover, in a mouse model, the wound that had not been exposed to CW microbial communities trended towards normal healing [36]. These results show that human CW microbiota is viable and maintains its ability to reestablish itself and infect another host in the context of permissive and optimal environmental conditions [36].

With respect to possible microbial predictors of wound healing, Verbanic et al. found that facultative anaerobes were significantly correlated with ulcers that did not heal within a six-month period, especially the *Enterobacter* genus [37]. Consequently, this finding suggests that an increased presence of facultative anaerobes may represent a negative prognostic factor concerning the CW microbiome, which may be due to the spectacular ability of these particular communities to adapt to a variety of metabolic microenvironments [37].

In a study from 2011, Tuttle et al. analyzed debridement samples in search for associations between venous insufficiency leg ulcer healing and bacteria [38]. They found that wounds that had not healed at six months showed an increased bacterial abundance and diversity [38]. Compared with those that did heal, *Pseudomonas* and *Actinomyccetalis* were significantly increased in ulcers that did not heal at six months [38]. Another finding was that *Bacteroidales* was increased in unhealed wounds and though it was trended toward, it did not reach significance [38]. The explanation behind this trend was thought to be the small sample size of only 10 probes [38].

A recent study from 2021 by Loomis et al. aimed to assess the influence of both individual taxa and microbial communities on cutaneous healing processes using human skin equivalents [39]. Pathways and key genes influenced by skin microorganisms were evaluated through transcriptomics analysis, while histological analysis was also used for a more detailed description of the impact on skin processes [39]. Tissues treated with either *Micrococcus luteus* or a mixed bacterial community showed a significantly reduced thickness of the human skin model [39]. Moreover, tissues treated with the *Corynebacterium* spp., *Micrococcus luteus*, or mixed bacterial community had a significantly reduced number of proliferating cells [39]. The presence of a microbiome on a human skin equivalent (a 3D tissue model) resulted in alterations of the gene expression, particularly genes involved in proliferation, apoptosis, and in the formation of the ECM [39].

In an experimental study from 2013, Pastar et al. raised awareness on the interaction between *Staphylococcus aureus* and *Pseudomonas aeruginosa* with the host in polymicrobial wound infections [40]. In their research, the USA300-0114 methicillin-resistant *Staphylococcus aureus* (MRSA) and *Pseudomonas aeruginosa* isolates were used for in vivo and in vitro experiments [40]. A porcine partial thickness wound model was used to elucidate the synergistic effects of *Pseudomonas aeruginosa* and USA300 on the healing process [40]. Results showed that *Pseudomonas aeruginosa* had the ability to inhibit USA300 growth in vitro while in vivo, both species co-existed in CWs [40]. In the presence of *Pseudomonas aeruginosa*, an increased expression of the USA300 virulence factors α-hemolysin and Panton–Valentine leucocidin was observed [40]. Moreover, re-epithelialization was delayed in case of the mixed-species infected wounds, particularly through suppression of the keratinocyte growth factor 1 (KGF-1) [40]. These studies brought evidence for the interactions between bacteria within mixed-species biofilms in vivo and for the contribution of virulence factors to the increased severity of polymicrobial infections in non-healing wounds [40].

**Table 1:** Bacterial species in the wound microbiome linked to wound healing.

Type of Chronic Wound	Microbiome Particularities
Diabetic foot ulcers	*Streptococcus* spp. [20,30] *Staphylococcus aureus* [20,30] *Staphylococcus epidermidis* [20,25] *Enterococcus* spp. [20] *Peptostreptococcus* spp. [20] *Bacteroides* spp. [20] *Prevotella* spp. [20] *Pseudomonas aeruginosa* [20] *Corynebacterium* [27,29,30] *Serratia* [28] *Peptostreptococcus* [28] *Peptoniphilus* [28] *Peptoniphilus* [29] *Anaerococcus* [29] *Anaerococcus* [30] *Curvibacter* spp. [41]
Venous leg ulcers	*Staphylococcus* [26] *Pseudomonas* [26] *Bacteroides* [26] *Serratia* [26] *Corynebacterium* [26] *Anaerobes* [26] *Klebsiella pneumoniae* *Escherichia coli* *Morganella morganii* *Porteus mirabilis* *Citrobacter freundii* β-hemolytic streptococci of group G
Pressure ulcers	Firmicutes [32] Proteobacteria [32] Actinobacteria [32] *Staphylococcus epidermidis* [32] *Corynebacterium striatum* [28,32] *Finegoldia magna* [32] *Streptococcus agalactiae* [28] *Pseudomonas aeruginosa* [28]

The wound microbiome may exert beneficial effects on the healing process [20]. Research in experimental models has distinctly shown the advantageous impact of certain gut microbiota members on regulating systemic inflammation, potentially affecting wound healing beyond the gastrointestinal tract [20]. For instance, researchers monitored wound healing subsequent to surgical skin incision and suture in both germ-free and conventionalized mice [20]. Initially, conventional mice exhibited greater tensile strength in the wound, along with higher levels of hydroxyproline concentration in the surrounding tissue compared to germ-free mice [20]. In another investigation involving dermal wounds in rats, the inoculation of wound sites with *Pseudomonas aeruginosa* PA01 expedited re-epithelialization, epidermal cell proliferation, and neo-vascularization, alongside the increased local infiltration of neutrophils and TNF production [20]. Treating these rats with antibodies targeting neutrophils or TNF resulted in a significant reduction in the wound healing response [20]. Additional experimental studies have highlighted a similar favorable effect of low-level wound colonization, even by potentially pathogenic microbes, contingent upon the degree of colonization and the type of wound [20].

Although the exact role of the microbiome on wound healing and outcomes has yet to be unveiled, advances in research are promising [19]. Therefore, studies focusing on the clinical correlation between the microbiome and wound repair, as well as research in the field of nonbacterial wound microbiomes (such as mycobiome and virome) may lay the foundation for developing novel methods for manipulation of the wound microbiota in order to promote healing, as is the case for probiotics or bacteriophage therapy [19].

## 4. Temporal Dynamics of Chronic Wound Microbiome under Treatment

The temporal dynamics of the CW microbiome is another issue of concern. Although the microbiome characterization by DNA sequencing may help in tailoring antibiotic treatments for wound biofilm infections, and therefore offer superior results compared to standard therapy, there is still scarce information on the temporal dynamics of bacterial communities that may also influence therapeutic response [42].

Tipton et al. conducted an experimental study on specimens obtained in three sampling points from 167 patients with CWs [42]. The results showed a significant relationship between the period of time between each sampling and the community similarity [42]. Communities commonly transitioned from *Staphylococcus*- or *Pseudomonas*-dominated states into a more variable state at different sampling points [42]. Even though low abundant bacterial species are often overlooked, the results showed that these species were frequently responsible for wound infection [42]. This study supports the idea that compositional shifts in microbial communities should be taken into consideration during the course of the treatment, as well as the importance of low abundant species to optimize biofilm-based wound management [42].

A case report from 2015 by Sprockett et al. followed the interesting dynamics of the bacterial load during wound healing in a patient with a chronic venous leg ulcer over 15 treatments [43]. By DNA-based methods, the authors revealed that the microbial burden of the wound is indeed surprisingly dynamic, with changes in the community structure of the wound’s microenvironment and the bacterial load, which are strongly linked with wound expansion, healing process, and antibiotic therapy [43]. Results showed that, initially, the bacterial diversity decreased due to antibiotic therapy; however, after the patient had finished his antibiotic treatment, it increased for a period of two weeks [43]. After two weeks, when the wound had begun its healing process, a decrease in bacterial diversity was observed yet again [43]. Swab samples also showed that the bacterial load seemed to be higher in specimens taken from the central part of the wound rather than the edges [43].

The clinical outcomes of a CW are associated with the temporal dynamics of the wound microbiota, as shown by Loesche et al. in a prospective, longitudinal cohort study from 2017 on 100 subjects with DFUs [30]. Specimens were collected at the baseline (initial presentation) and resampled every two weeks until the DFU healed, an amputation occurred, or the 26 weeks of follow-up had ended [30]. All patients underwent wound debridement and offloading [30]. Results showed that of the 100 patients, 31 developed an infection-related complication, which was defined as either osteomyelitis, wound deterioration, or amputation [30]. Furthermore, DFUs with a dynamic microbiota healed faster than the wounds with less dynamic microbiota [30]. Effects of antibiotic therapy on the temporal stability of DFU microbiota has also been evaluated and the following compelling findings were observed: antibiotics prescribed for the study ulcer produced an increased community disruption compared with antibiotics prescribed for infections not involving the CW, such as sinus infection, upper respiratory tract infection, or urinary tract infection [30]. Furthermore, there were some cases in which a wound complication was described during the same period an antibiotic agent was administered [30]. Research showed that although both wound complication and antibiotic therapy disrupted the microbiota community, the larger effect on this phenomenon was seen for antibiotics [30].

In 2020, Pang et al. brought attention to the theory of human–microorganism mutualism as a possible mechanism for delayed healing in CWs [44]. This theory implies that the harmony with microorganisms aids in assuring a protective barrier in the skin or gastrointestinal system, while it also helps modulate the immune system [44]. Furthermore, the microbiome acts as an effective biological barrier by killing pathogens or through direct competition with potentially harmful microbial species [44]. Modifications in the physical state and in the environment are key factors responsible for alterations of the human microbiome and occurrence of disease, which is the case for chronic non-healing wounds [44]. The authors believe that the therapeutic approach to CWs should focus on helping the microbiome to reach a balanced state with the host, rather than applying rigorous sterilizing methods [44].

One of the very first steps in the management of wound care is represented by careful debridement. A comparative study from 2020 by Verbanic et al., which included 20 outpatients with longstanding ulcers aimed to assess the short-term effect of wound debridement on the local microbiome of CWs [37]. After one session of sharp debridement, the wound microbiome was not significantly altered, showing that there were no differences between the microenvironment of the original ulcer surface compared to that of the one exposed to a one-time debridement procedure [37].

In an experimental study from 2017, Kalan et al. determined whether culture-independent molecular methods may be used to identify the composition of the microbiome in CWs and measure it over time when topical antimicrobial dressings are used locally to reduce the bacterial load [41]. In this study, patients with wounds of more than six weeks in duration, and who are not taking systemic antibiotic therapy, were enrolled [41]. Immediately after the routine debridement specimens were collected, as well as swabs from the wound bed, a dressing containing silver oxynitrate was applied [41]. To assess the microbiome, next-generation sequencing of the microbial 16S rRNA gene for each specimen was used [41]. Results showed that there were distinct bacterial communities between the debridement and swab samples [41]. In the pre-treatment samples, results showed that the relative abundance of *Curvibacter* spp. was further increased in diabetic patients compared with non-diabetic patients [41]. The proportion of *Staphylococcus aureus* was higher in non-foot/leg ulcers than in foot or leg wounds [41]. Regarding the temporal changes in microbial communities of the specimens collected after wound dressing application, the relative proportions of *Staphylococcus* spp. shifted compared to the pre-treatment evaluation [41]. An increased abundance of *Staphylococcus* spp. for both swab and debridement samples in foot and leg wounds was found, while a marked decrease in non-foot/leg ulcers was observed [41]. Non-leg ulcers displayed an increased microbiome diversity than foot and leg ulcers for both swab and debridement specimens [41].

## 5. Host Factors Impacting the Composition of CW Microbiota and Healing

### 5.1. Host Genetics

In the last years, host genetics has been regarded as an important determinant in the composition of individual-associated microbiomes. In a review from 2019, Awany et al. stated that progress in next-generation sequencing technologies may enable the understanding of the interaction between microbial communities and host genetics [45]. Microbiome genome-wide association studies (mGWAS) allow for the discovery of the patient’s genetic variants, which leads to variability in the composition and function of the microbiome of every individual in both health and disease [45]. The role of human genetics has emerged as influential in determining interpatient differences in the microbiome [45]. Human genes may influence health directly or by promoting a beneficial microbiome [45]. Associations between microorganisms, microbial genes, and human genes are considered to be consistent between human populations [46].

As shown anteriorly, at the present moment, the pathogenesis of delayed wound healing is complex and not thoroughly understood. Nevertheless, advances in molecular biology techniques have allowed for insight into the potential association between the host’s genetics and the composition of a CW microbiota, and overall, of the proper healing process [47]. In a two-cohort microbiome-genome-wide association study from 2020, Tipton et al. brought attention to the importance of patient-specific processes that lead to the evolution of a CW by identifying patient genomic loci correlated with the diversity of the microbiome of a CW [47]. Investigations revealed that the inter-patient variation in relative abundance of *Pseudomonas aeruginosa* and *Staphylococcus epidermidis*, widely known as two key pathogens in longstanding wounds, were explained by the alternative *ZNF521* and *TLN2* genotypes [47]. Moreover, the composition of a genotype-associated microbiome was also related to the healing process, therefore it was observed that wounds with lower diversity microbiomes had prolonged durations until closure [47]. In a study from 2019, Deusenbery et al. studied the human macrophages response to several microbial supernatants from DFUs [48]. Human monocyte-derived macrophages obtained from four donors were cultured for 24 hours in media, which were conditioned by both bacteria and fungi (*Corynebacterium amycolatum*, *Pseudomonas aeruginosa*, *Staphylococcus aureus*, *Corynebacterium striatum*, and *Candida albicans*) that were isolated from the DFUs of six subjects [48]. Results showed that macrophages have the ability to respond to secreted factors from microorganisms by the upregulation of inflammatory markers, with the effects being dependent on the monocyte donor [48]. The above-mentioned genotypic effects may be the answer for these patient-specific immunological responses in the context of the same microbial exposure [47]. The theory which implies that the patient’s own genotype may predispose them to infection by certain specific species and may also determine the composition of the microbiome in the CW shows the selection by host genotype as well as species interactions in shaping the CW microbiome for every individual [47].

In a review from 2018 by Weissbrod et al. on host genetics and microbiome associations, the authors addressed three main issues which may aid in the accuracy of future research based on genome association studies, and included the following: firstly, the need for adopting a uniform data and standard reporting formats to assure replication and meta-analysis; secondly, implementing rigorous statistical criteria to decrease the number of false positive results; and thirdly, considering the microbiome of the host and the individual as completely independent entities, rather than evaluating single nucleotide polymorphism and different taxa separately [49].

### 5.2. Host Immunity

The immune response to biofilm infections is complex in the way that these bacterial aggregates may both overstimulate and suppress the immune system [50]. These complete opposite responses are influenced by several factors such as the species composition, the anatomical site of the infection, the immune status of the host, and the specific antigens that the immune cells interact with [50]. The immune system recognizes many bacterial factors, some of them being associated with the bacterial cell (e.g., cell wall, lipopolysaccharide, and flagella) or secreted [50]. Some secreted factors may be found in the biofilm matrix and some of them are exoproducts which allow other functions [50]. These particular factors are all immunogenic [50]. The extracellular polymeric substance of the biofilm is mainly composed of water, lipids, biosurfactants exopolysaccharides, exoproteins, and extracellular DNA, among others [50]. Each one of these molecules plays an important part in the overall functionality of the biofilm, collectively comprising the adhesive, structural, metabolic, and protective properties typical to biofilm pathogens [50]. Furthermore, they are important for the immune reaction to biofilm infections generated by the host, particularly for immunogenesis and immunomodulation [50]. In addition to the immune response generated towards the biofilm-forming bacteria, it is considered that the immune system generates a response mainly against three principal extracellular polymeric substance components, which include the following: the extracellular DNA, exopolysaccharides, and exoproteins [50]. Consequently, in the context of a biofilm infection, the immune system of the host plays an important part in terms of both immunogenesis and immunomodulation [50].

The innate immune response is believed to be an aberrant one, with CWs being enclosed in a persistent, chronic, inflammatory state [51]. Pathogens and their components directly promote the influx of both neutrophils and macrophages, and since they are a well-known source of proteases, it appears that the microenvironment in a longstanding wound is highly proteolytic [51]. Moreover, MMP activity is upregulated, and MMP-inhibitor activity is significantly downregulated [52]. Oxidative stress enhances chronic inflammation in longstanding, non-healing wounds [53]. Leukocytes are known to be the major source of reactive oxygen species (superoxide anion, singlet oxygen, hydrogen peroxide, hydroxyl radicals), rendering the microenvironment of the wound highly pro-oxidant [53]. Apart from directly damaging the structural proteins of the ECM, reactive oxygen species may also alter the transcriptional regulation and signaling pathways of proinflammatory chemokines and cytokines [54]. Consequently, provisional matrix components, mediators of repair, and growth factors are inactivated by proteolytic cleavage [53]. Although leukocytes may be plentiful at the site of the CW, their functions, namely chemotaxis, phagocytosis, and bactericidal activity, are markedly diminished, a finding mostly seen with diabetic ulcers [55]. Therefore, the wound becomes even more susceptible to bacterial colonization and infection [54]. Elucidation of the interaction between the wound microbiome and the innate immune system may lay the foundation for the development of effective non-antimicrobial treatment alternatives [54]. Potential therapeutic options may involve normalizing wound microbiota composition through either the promotion of commensal species or inhibition of pathogenic bacteria as an inexpensive and noninvasive alternative for the management of CWs [54]. Concerning the host relationship with the wound microenvironment, inflammatory factors may represent a therapeutic target aimed at controlling the persistent inflammation and to modulate the behavior of the CW-associated pathogenic bacterial populations [54].

In a recent experimental study from 2019, De Oliveira et al. aimed to evaluate the activity of the epidermal growth factor (EGF) versus platelet-rich plasma (PRP) against CW microbiota (mostly consisting of *Staphylococcus aureus* and *Pseudomonas aeruginosa*) [56]. Other objectives were represented by providing an in-depth description of the presence of infection in EGF- and PRP-treated wounds and to analyze the susceptibility profiles of the *Staphylococcus aureus* and *Pseudomonas aeruginosa* isolates [56]. A total of 43 specimens collected with swabs were obtained from 31 patients treated with EGF and PRP in an outpatient clinic of a hospital, of whom 41.9% had been treated with EGF and 58.0% with PRP [56]. A total of 10 out of the 43 specimens were identified as *Staphylococcus aureus*, 60.0% of which were isolated from PRP-treated wounds [56]. Among the 33 *Pseudomonas aeruginosa* specimens, 66.6% were isolated from platelet-rich-plasma-treated wounds [56]. Concerning antimicrobial susceptibility, one strain obtained from an epidermal-growth-factor-treated wound was identified as MRSA [56]. With respect to *Pseudomonas aeruginosa* isolates, one specimen obtained from a patient treated with the epidermal growth factor was multidrug resistant [56]. Finally, the authors found a significant difference between the 1st and 12th week, concerning wound infection improvement seen in the subjects treated with PRP (*p* = 0.0078) [56].

In a review from 2019, Brazil et al. described the crosstalk between innate immune cells and epithelial cells during the wound repair process [57]. In response to damage, epithelial wounds are able to repair themselves due to the integration of epithelial responses with that of both the resident and the infiltrating immune cells, mostly monocytes/macrophages and neutrophils [57]. Chronic non-healing wounds develop when this complex interplay is compromised, which contributes to the morbidity and mortality of many diseases [57]. A solution for this matter may require a better understanding of crosstalk between immune and epithelial cells during wound repair, so that novel strategies to treat debilitating complications may be developed and applied in clinical practice [57]. Therapeutic strategies for enhanced wound healing through increased macrophage recruitment, as well as orientation of macrophages toward a pro-repair state, and methods to increase macrophage recruitment have been investigated [57]. An example is represented by the direct injection of interleukin-1β (IL-1β)–activated macrophages into murine skin wounds [57]. An increased production of vascular endothelial growth factor C (VEGF-C), which improved wound repair, was observed at the wound site [57]. An increase in wound-associated macrophages and improved wound healing was seen when applying local granulocyte–monocyte colony stimulating factor (GM-CSF) to dermal wounds [58]. The methods aimed at enhancing either production or delivery of molecules which may promote tissue repair have also been investigated, primarily due to the complexity of the biomolecular environment found within chronic ulcers [59]. For example, it has been found that glutamine-loaded hydrogels were able to promote wound closure and re-epithelialization in wounds [59]. It appeared that collagen deposition was consistent with the increased activity of macrophages that were activated alternatively [59]. Contrarily, strategies aimed at inhibiting the alternative macrophage activation may reduce the unrestrained collagen deposition and therefore aid in preventing fibrosis and scarring [60]. The previously described innovative and potential therapeutic methods targeting macrophages (either their activity or orientation towards promoting wound healing) may represent promising solutions for a modern and novel curative approach in the management of longstanding wounds [61].

### 5.3. Microbiome Gut–Brain–Skin Axis

A review from 2019 by Vojvodik et al. sheds light on the complex interplay between gut microbiome and the modification of immune balance in skin diseases [62]. In this publication, the authors define and describe what is known as the psycho-neuro-endocrine-immunology (PNEI) approach [62]. PNEI is a bidirectional crosstalk, an interdisciplinary concept between the immune system and the psycho-neuro-endocrine systems, with an impact on immune response [62]. The immune system may also influence the neuro-endocrine response and this complex relationship is mediated by several neuropeptides, different hormones, growth factors, and signaling molecules [63]. An example of the PNEI concept may be the relationship of the skin and gut with other tissues and organs [62].

In a review from 2020, Hadian et al. hypothesized that since the composition of the gut microbiome may influence the brain and consequently the behavior and cognitive function, perhaps cutaneous dysbiosis found in CW infections may also influence behavior and cognition [64]. Moreover, while the gut microbiome may directly alter the cutaneous microbiome, the skin may also alter the gut microbiome [65]. Therefore, a novel bidirectional signaling concept emerged, the “skin–gut axis” [65].

Both the skin and the gut are colonized by specific microbial strains, exert tolerance towards the commensal microbiota, are constantly exposed to antigenic charge, and have a great number of neural and vascular structures [62]. Therefore, the already notorious gut–skin axis and gut–brain axis may be merged into a larger concept, which is the” gut–brain–skin axis”, a complex network modulated by multiple neuro-hormones, neuropeptides, cytokines, and other signaling molecules [66,67].

Modifications in the intestinal microbiome have been associated with a broad range of skin diseases such as acne, psoriasis, and atopic dermatitis [68,69]. Nevertheless, similar evidence and correlations have not yet been established between microbiomes of the gut and chronic skin wounds [64].

### 5.4. Stress Hormones

Advances in the field of molecular biology, together with bioinformatic and genomic approaches, have enabled an in-depth characterization of both epidermal and dermal microbiomes [70]. Nowadays, this technology aids in identifying statistical associations between certain microbiome phenotypes and outcomes with respect to cutaneous healing and response to wound infection [70]. Skin pathologies, metabolic diseases, perceived stress, anxiety, and depression may act as triggers, influencing the host–microbiome interaction and wound healing [70].

Stress-induced mediators, such as glucocorticoids, catecholamines, and acetylcholine may impair wound healing, increasing the risk for wound infection [70]. The three principal pathways of the stress response include cholinergic stimulation via acetylcholine, catecholamines via adrenergic stimulation, and glucocorticoids via activation of the hypothalamic-pituitary-adrenal (HPA) axis [70]. Although a multitude of conditions affect the interplay between the host and stress, certain diseases predispose patients to the development of altered stress responses, impaired wound healing and chronicization [71]. Diseases like peripheral vascular disease, diabetes, and immune suppression, as well as advanced age are commonly associated with an altered stress response, impaired wound healing, and chronicity of wounds [71,72]. To identify the microbial phenotypes that influence the immune responses required for an adequate healing process, there is an imperative need for a description of the pathways by which stress-induced hormones impact the bacterial proliferation and metabolism within the CW microenvironment [70].

A recent review from 2021 by Luqman and Gotz shows that the skin microbiota and adrenaline not only share an interplay, but also play an ambivalent role in wound healing [73]. A stress response triggered by a skin injury leads to an increase in both local and systemic levels of stress hormones such as cortisol and adrenaline [74,75]. Adrenaline is particularly essential at the first stage of the healing process, for both the homeostasis and inflammatory phases [73]. Nevertheless, prolonged increased levels of adrenaline results in a delay in the development of further stages of wound healing, in the proliferative and maturation phases [76].

After the skin is injured, epithelial cells produce and release adrenaline (epinephrine) as a stress reaction, which further activates the β2-adrenergic receptor (β2-AR), resulting in an impaired migration of keratinocytes and altered re-epithelization [73]. Moreover, adrenaline not only affects the functionality of keratinocytes, but also promotes the growth and virulence of bacterial pathogens [73]. Trace amine-producing commensals, such as *Staphylococcus epidermidis* or members of the *Macrococcus* and *Firmicutes* genera, may subvert the negative effects of adrenaline and encourage proper wound healing [73]. Even though these bacteria are part of the normal cutaneous microbiota, their proportion may vary from one individual to another [73]. It is hypothesized that trace-amine-producing bacteria may be a promising therapeutic alternative in CW management [73].

Nowadays, it is well-recognized that keratinocytes have the ability to produce and release stress hormones [75]. During the acute phase of wound healing, cortisol production in the epidermis is rigorously controlled [75]. Furthermore, a key molecule in the pathway of cholesterol synthesis, namely farnesyl pyrophosphate (FPP), binds the glucocorticoid receptor (GR) and activates it [75]. In addition to that, keratinocytes begin to express beta-2-adrenergic-receptor (β2AR), the receptor for adrenaline (epinephrine) [75]. The main issue resulting from these series of events is that migratory rates of keratinocytes are remarkably lowered by cortisol, farnesyl pyrophosphate, adrenaline, and other β2AR agonists, pointing at their role in the inhibition of proper re-epithelization [74,75,77]. As a consequence, there is no progression through the normal steps of healing and a continuous state of inflammation is created in the wound microenvironment [75].

Therefore, in the near future, a therapeutic approach may be represented by agents who block either the enzymes and/or receptors involved in the production, release, and function of the anteriorly mentioned stress hormones [75].

Despite detailed characterization of the skin microbiome provided by high-throughput genomic technologies, the correlation between the cutaneous microbiome and stress-induced hormones on the development of the skin microbiome during wound healing has yet to be further studied [70]. Moreover, the characterization of the host–microbial profile, which is unique for each individual, and taking into consideration stress molecules and microbial markers, may aid the identification of patients at risk for developing longstanding wounds or infections, thereby allowing for an earlier intervention [70].

## 6. Probiotics and Prebiotics in Wound Healing

The complex interplay between the human body and its inhabiting microflora along with dietary habits, hygiene, and other external factors contributes to the pathogenesis of many dermatologic conditions (wound healing, psoriasis, acne, rosacea, atopic dermatitis, seborrheic dermatitis, body malodor, and others) [21,78,79,80,81]. Commensals and symbionts along with pathogens on human skin have important roles in the inflammatory response, which highlight several novel strategies to treat non-healing wounds [21]. The constant failure of antibiotic therapy, to some extent due to the formation of biofilms, has challenged scientists to look after alternative therapeutic approaches to modulate the microbiome, mainly comprising prebiotics and probiotics (due to their ability to displace pathogens) and bacteriophages [21,78,82].

As a result, a quick increase in the medical use of probiotics and prebiotics has been recently noted, as they can act as antimicrobials with an exceptional safety profile [79,81,82]. Although their mechanism of action is not yet fully elucidated, probiotics and prebiotics target microbiota imbalances and modulate the immune system [79,81,82]. Synbiotics, a combination of pre- and probiotics, have a synergistic effect on the gut microbiota [79,80]. These immune modulators already proved to be efficient in inflammatory skin diseases, but their role in wound healing, as well as other dermatological conditions still requires further investigations [82]. Although they are promising agents, probiotics and prebiotics need to pass larger trials before being implemented in the clinical practice [78,79,82].

Probiotics are live microorganisms which provide certain benefits to the host when administered in adequate amounts as part of preventative or curative treatments [78,79,80,82,83].

*Lactobacillus*, *Bifidobacterium* and *Enterococcus* (also found in the intestinal microbiota) are the most frequently used probiotics and can be found in fermented milk products, pills, powders, and topical preparations [17,78,79,80].

In wound healing, probiotics may be beneficial due to various roles including the following: they produce bacteriocins and organic acids with antimicrobial properties; compete with *Staphylococcus aureus* in keratinocyte attachment; prevent the increase in elastase, interleukins-1B and -10; prevent the decrease in Langerhans cells induced by ultraviolet (UV) radiation; exhibit antioxidant properties and decrease UV skin damage; enhance skin hydration and dermal thickening; and promote healing via modulation of the inflammatory response and restraining pathogen colonization [78,79,81,82]. Moreover, probiotics, due to their interleukin-10 mechanism, have been linked to healthy skin and hair in mice [82].

Probiotics’ mechanism of action comprises their effects on both innate and adaptive host immune responses [80].

Probiotics have the ability to disrupt biofilms by modulating neutrophils activity via IL-8 levels regulation [82]. By reducing both necrosis and apoptosis of neutrophils in CWs, it may enable tissue repair due to a more efficient phagocytosis in wound debridement and a decrease in the bacterial load [82].

When administered orally, probiotics contribute to gut immune homeostasis by their interaction with epithelial cells and their influence on both innate and adaptive immune responses via similar pathways as commensal bacteria [80]. They enhance the epithelial barrier function through interactions with Toll-like receptor 2 (TLR2) and inhibit pathogen growth by predominantly modulating dendritic cells (DC) and T regulatory cells, rather than T helper responses [80]. As a consequence, they influence epithelial cell signal transduction pathways and cytokine production, suppressing inflammatory responses [80]. Individual bacterial strains may modulate IL-10 and IL-12 production and the expression of maturation and co-stimulatory markers by DCs [80].

*Bifidobacterium* strains seem to enhance IL-10 production, downregulate co-stimulatory molecules (CD80, CD40), and decrease IL-10 dependent interferon-gamma (INF- γ) production [80].

The effects of *Lactobacillus* seem to vary significantly between strains. They may induce, downregulate or exhibit no effect on IL-10 production; induce T regulatory cell production and tolerance via the generation of semimature DCs; inhibit T cell proliferation; stimulate Th1 cytokine production, increase Th2 responses, or induce a mixed Th1/Th2 response [80].

Nevertheless, research data regarding the use of probiotics in cutaneous wound healing are quite limited but with promising outcomes [78].

In vitro, *Lactobacillus plantarum* was found to inhibit the production of elastase, biofilm formation, and acyl homoserine lactone by *Pseudomonas* spp. [21].

In a meta-analysis of animal studies encompassing six investigations (five involving topical application and one utilizing an oral route), it was determined that the use of probiotics had a positive impact on expediting the initial phases of skin wound healing [78]. Within this review, it was noted that sterile kefir extracts, which contained filtered supernatants from kefir culture fermentation, demonstrated superior effectiveness compared to bacteria and yeast [78].

In a study on mice with burn wounds performed by Yu et al., topical *Lactobacillus plantarum* inhibited *Pseudomonas aeruginosa* colonization, promoted tissue repair, and enhanced phagocytosis [81].

Poutahidis et al. underlined that supplementing the intestinal microbiome with lactic acid bacteria (e.g., *Lactobacillus reuteri*) could accelerate cutaneous wound healing in animals via the upregulation of the neuropeptide hormone, oxytocin [84,85]. As a result, both the cutaneous and gastrointestinal microbiome participate in the healing process of wounds [21].

Other studies on rabbits with infected full-thickness wounds concluded that nitric-oxide-producing probiotic patches accelerated healing and reduced the bacterial load [82].

Honeybee lactic acid bacteria applied on chronic equine wounds seemed to promote wound healing and inhibit the growth of pathogens in vivo [21].

In patients with burns or chronic VLUs, other research studies showed that probiotics may be able to decrease the bacterial load and thus promote wound healing [81,82].

Moreover, in rabbit models with *Pseudomonas* infected burn wounds, *Lactobacillus plantarum* lowered collagen accumulation and thus decreased the extent of scarring [80].

Peral et al. concluded that topical application of *Lactobacillus plantarum* on patients with burns had the same efficacy as standard silver sulfadiazine in lowering the bacterial load, promoting granulation tissue development, acceleration of wound contraction, and thus wound healing [86]. Due to the reduced number of patients in the analyzed group (80), scientists could not reach statistical significance and thus additional studies are still required to discover whether *Lactobacillus plantarum* is a beneficial therapeutic alternative for wound healing [86].

Topical treatment with probiotics containing *Lactobacillus plantarum* contributed to a decrease of about 90% of the wound area in 43% of diabetic patients with chronic VLUs, and in 50% of non-diabetic individuals, and to a reduction in colony-forming units via regulating IL-8, phagocytic cell, and fibroblast recruitment in addition to improved glycemia levels [21,81,82].

Oral probiotics containing *Lactobacillus* spp. also proved to be effective in managing chronic diabetic ulcers, resulting in favorable outcomes such as decreased ulcer size and reduced levels of inflammatory markers [81].

Skin commensal *Propioniferax innocua* seemed to disrupt established pre-formed biofilms as a result of its adaptation to the skin environment and further superior resilience [81].

*Staphylococcus epidermidis* was able to produce antimicrobial compounds which selectively target *Staphylococcus aureus* and *Staphylococcus pyogenes* and suppress skin inflammation through lipoteichoic acid [21,81]. Also, *Staphylococcus caprae* seems to have antimicrobial activity against methicillin-resistant *Staphylococcus aureus* and inhibit its colonization in mouse models [81].

Prebiotics are non-digestible carbohydrates, typically plant- or food-derived molecules, which can selectively trigger the growth and/or activity of probiotic bacteria and as a result, can improve health [78,82]. In order to be considered a prebiotic, an ingredient should resist breakdown by both mammalian enzymes and gastrointestinal absorption and be fermented by the microbiota [82].

Indigestible oligosaccharides are commonly used compounds, inulin-type fructans (ITF) and galacto-oligosaccharides (GOS) being the most available ones [78,82]. Their mechanism is based on promoting the growth of lactic-acid-producing bacteria and bifidobacteria in the intestinal tract, leading to comparable benefits to those observed when directly ingesting these microorganisms as probiotics [21]. Furthermore, prebiotics have an improved delivery, safety status, and affordability, and thus represent complementary or alternative choices to probiotics [82].

Moreover, postbiotics such as short chain fatty acids (acetic, butyric, and propionic acid) produced by skin commensals possess anti-microbial properties and it may be useful to incorporate them into the treatment of cutaneous wounds [3]. Either injected or topically applied, short chain fatty acids inhibit cutaneous inflammation by promoting skin T regulatory cells in a histone-acetylation-dependent mechanism [21].

Bacteriophages (phages) are viruses which are able to infect bacteria, highly specific to bacterial species or even to particular strains, that rapidly reproduce, transfer DNA, and lyse cells [78]. They are considered helpful tools to alter or re-establish the equilibrium of the microbiota [78]. Bacteriophages may also improve the access of antibiotics in the targeted area, useful in the management of various dermatologic bacterial infections [78]. Unlike antimicrobials, they can reach specific sites inside the human body and further replicate to the demanded amount, relying on the bacterial population [78]. There are presently a lot of approaches to phage therapy such as the direct use of the phage lytic/lysogenic cycle; the use of phage enzymatic compounds to attack pathogens; the use of phages to disrupt bacterial biofilms; the use of phages to integrate into the human genome; and the introduction of antibiotic sensitive genes useful in decreasing antibiotic resistance [78].

Phage therapy is currently under investigation for its role in treating wounds (ulcers), burns, and skin-related infections [78]. In vitro studies showed promising results of lytic phages isolated from *Cutibacterium acnes* in clearing areas from bacterial lawns [78]. Various phages from the *Siphoviridae* family, isolated either from skin scrubs or swabs samples from patients, were assessed for their potential activity against *Cutibacterium acnes* [87]. Positive in vivo outcomes have been observed, where creams and bandages containing both phages and antibiotics can be externally administered to the afflicted skin [78]. Moreover, an additional potential use of phages in wound prophylaxis against bacterial infection is implemented in the clinical practice in Georgia [78].

Because of the lack of confirmatory studies, there still are some concerns in terms of phage therapy bioavailability and elimination rate [78]. Additionally, the efficiency of phages in treating intracellular pathogens has not yet been settled [78]. Resistance towards therapy has also caused some concern due to “pseudolysogeny” (no integration of phages genome in the bacterial genome and thus no lysis) [78].

Other potential therapies to enhance wound healing include biofilm disruption agents such as quorum sensing inhibitors, engineered synthetic peptides with unique anti-biofilm properties, or predatory bacteria (*Bdellovibrio bacteriovirus*), which seemed to reduce biofilm development by effectively destroying large numbers of pathogens [21].

A future direction in improving therapeutic approaches for non-healing wounds also includes the use of mesenchymal stem cells due to their role in suppressing inflammation, reducing scar formation, stimulating angiogenesis, all leading to promising results in CW healing [88].

A better understanding of the pathogenesis of CW and of the host–microbiome interplay could contribute to a tailored therapeutic manipulation of microbiome (in composition, functions, others) in order to enhance healing. Moreover, it is necessary to discover which prebiotics, probiotics, or synbiotic treatments can be used to reach a normal and balanced microbiome composition [78,79]. Certainly, it would be of great interest to study the possible modalities of delivery of the prebiotics, probiotics, synbiotic, and bacteriophages which will lead to significant scientific progress in this field [78].

## 7. Future Perspectives in the Diagnosis and Treatment of Chronic Wound Infections

In spite of the several extensive studies conducted over the past few years, delayed healing still represents a worldwide burden [3,14]. One approach to achieve information on the mechanisms of disease progression and therapeutic response is the use of multiple high-throughput ‘OMIC’ modalities (genomic, proteomic, lipidomic, metabolomic assays), facilitating the discovery of potential biomarkers [2,10,14].

OMIC studies currently focus on establishing which is the appropriate combination of molecules that relates to the healing status [2]. It provides means to assess panels of biomarkers, which represent useful tools, to predict healing outcomes and develop novel targeted therapies [2]. As a consequence, recent scientific papers focused on the possible role of these biocompounds, which may predict healing outcomes, monitor disease progression or regression, quantify the therapeutic response, and possibly allow further personalized and cost-effective treatments [2,10,14,89]. There has also been a rather general focus on identifying both inflammatory biomolecules and those acting as normal modulators in the chronic wound fluid (CWF) through the healing process [2].

Molecular biomarkers represent a wide array of quantifiable biological substances that can be functional or non-functional, specific or non-specific, and can be used as indicators of a normal biological processes, pathogenic processes, or pharmacologic responses to a therapeutic intervention [2,3,10,14,85,89]. Biological markers can be used in the whole spectrum of the affliction process and are produced either by the affected organ, as in the case of tumors, or by the human body as a response to certain conditions [10]. Based on the type of information they provide, chronic wound biomarkers are classified as follows: diagnostic (to guide debridement), predictive (patient outcome, therapeutic benefits), prognostic (disease recurrence, progression), monitoring or indicative (wound healing status, patient response to therapy by serial measurements), and biomarkers of safety or pharmacodynamic markers [10,14,89,90].

Currently biologic markers can be identified by measurement in swabs, wound microbiota, tissue specimens (β-catenin and c-myc), wound fluid (MMPs, interleukins), and serum (procalcitonin and MMPs) [10,14,91]. Besides traditional tools (erythrocyte sedimentation rate (ESR), C-reactive protein and albumin levels) to assess both healing potential and wound infection, researchers have identified several potential biomarkers, including macrophages, neutrophils, fibroblasts, and cytokines (including TNF- α, interleukins (ILs), growth factors (GF), MMPs, TIMPs, etc.) [10]. An ideal biomarker should be safe, facile to use, and should not encounter any variations with gender or ethnicity [10]. Some of the most relevant ones will be thoroughly discussed in the following paragraphs.

Biomarkers also serve to achieve more informative therapeutic research by acknowledging the disease progression and the effects of therapeutical interventions [89]. There are diverse ways by which biomarkers lead to the development of novel therapies [89]. They represent highly sensitive and specific indicators of disease pathways and act as substitutes for diseases outcomes in clinical trials by predicting clinical risks or benefits [8]. For example, B-type natriuretic peptides are valuable biomarkers in the spectrum of heart failure, recently being associated with a higher risk of cardiovascular events when present in pregnant women with associated comorbidities [92].

Finding specific biomarkers for wound healing would represent a breakthrough in this particular field and also a major help in addressing delayed wound healing [3,10].

## 8. Conclusions

CWs are growing more prevalent, significantly impacting the patients’ quality of life by their long-term evolution and severe complications. A lot of factors should be taken into consideration regarding interference with delayed wound healing, which is a major public threat and a substantial burden to healthcare systems. Biofilm development is a significant virulence trait which enhances microbial survival and pathogenicity and has elaborated implications on the treatment of non-healing wounds. As a result, wound care involves increased costs and represents a substantial worldwide economic burden. Moreover, the development of antimicrobial resistance by the microorganisms that colonize the ulcers lead to an alarming epidemiological risk.

The human–microorganism mutualism theory may help us overcome these concerning issues and discover innovative treatment strategies.

OMIC approaches (proteomics, metabolomics, others) have contributed to the discovery of biocompounds that predict healing outcomes, help monitor the progression/regression of the disease, quantify the therapeutic response, and allow for personalized and cost-effective treatments. Discovering specific biomarkers for wound healing would surely represent a breakthrough in this field and a major help in addressing delayed wound healing.

The interplay between the human body and its inhabiting microflora may lead to different dermatologic afflictions. The microbiome has essential roles in dermatology, including as a therapeutic target. Human skin commensals, symbionts, and pathogens influence the inflammatory response, highlighting potential novel strategies to treat non-healing wounds. Although prebiotics, probiotics, and bacteriophages represent alternative therapeutic approaches able to modulate the microbiome and positively contribute to wound healing, long-term safety data and larger clinical trials are demanded to support their implementation in the clinical practice.

A better understanding of the pathogenesis of dermatologic disorders could contribute to therapeutic manipulation of the microbiome composition and the discovery of possible modalities of targeted compounds delivery, which will lead to significant scientific progress in this field.

## Data Availability

This review summarizes data reported in the literature and it does not report primary data.
